# Complete Genome Sequence of Soil Fungus *Aspergillus terreus* (KM017963), a Potent Lovastatin Producer

**DOI:** 10.1128/genomeA.00491-16

**Published:** 2016-06-09

**Authors:** Janakiraman Savitha, S. D. Bhargavi, V. K. Praveen

**Affiliations:** Department of Microbiology and Biotechnology, Bangalore University, Bangalore, India

## Abstract

We report the complete genome of *Aspergillus terreus* (KM017963), a tropical soil isolate. The genome sequence is 29 Mb, with a G+C content of 51.12%. The genome sequence of *A. terreus* shows the presence of the complete gene cluster responsible for lovastatin (an anti-cholesterol drug) production in a single scaffold (1.16).

## GENOME ANNOUNCEMENT

Lovastatin is a potent drug that is used for the treatment of hypercholesterolemia and was the first drug among statins which was approved by United States Food and Drug Administration (USFDA) in 1987. It acts as one of the competitive inhibitors of the enzyme hydroxyl methylglutaryl coenzyme A (HMG-CoA) reductase, which catalyzes the conversion of HMG-CoA to mevalonate during cholesterol biosynthesis (1). Various filamentous fungi such as *Monascus ruber, Penicillium citrinum, Paecilomyces variotii*, etc. are reported to produce lovastatin (2). However, a strain of *Aspergillus terreus* (ATCC 20542) is being used for commercial production. The genes responsible for lovastatin biosynthesis are *lovA, lov*E, *lov*B, *lov*C, *lov*F, and *lov*D which make a complete gene cluster (3).

In a previous study, we reported a tropical soil fungal isolate, *A. terreus* (KM017963), which produces a significant amount of lovastatin (4). This strain was grown in several agro-based natural media to select the best substrate for increased yield of lovastatin (5). Genetic and bioinformatic analysis of the whole genome of the lovastatin-producing soil isolate *A. terreus* (AH007774) revealed the presence of the lovastatin gene cluster (6, 7). Using the existing nucleotide sequence information and devising suitable primers, the target PCR amplification of the two important genes, *lov*E (1,512 bp) and *lov*F (749 bp), were successful in cDNA (7) and genomic DNA (unpublished data) of *A. terreus* (KM017963). Results of the above studies have categorically concluded that *A. terreus* (KM017963) is a potent lovastatin producer. In order to get further and deeper insight of our isolate’s lovastatin gene cluster, the whole-genome sequencing of *A. terreus* (KM017963) was performed, which further confirmed the presence of the lovastatin gene cluster.

The fungus *A. terreus* (KM017963) was cultured on Potato Dextrose broth at 28°C, pH 6.0 and incubated in a shaker at 120 rpm for 7 days. Genomic DNA was extracted using cetyltrimethyl-ammonium bromide (cTAB) (8). The quality and quantity of DNA was checked on 1% agarose gel and Nanodrop 2000 (A260/280), respectively. Further determination of DNA concentration was performed using a Qubit3.0 Fluorometer.

Whole-genome sequencing was performed using HiSeq2500. We constructed and sequenced a paired-end library to obtain filtered reads of 20,116,834. The high-quality reads were assembled using AbySS (version 1.5.2) and SSPACE (version 3.0). The average gene length was 1,945 bp. A total of 5,202 genes were predicted using Agustus (version 3.2.1). Reads (91.78%) were mapped to the reference genome with 96.88% coverage. A total number of 25,151 single nucleotide polymorphisms (SNPs) and 2,644 indels were discovered using the standard pipeline of SAMtools mpileup. The lovastatin gene cluster (AF141924.1 and AF141925.1) comprises a total number of 17 genes, out of which 3 genes were present in AF141924.1 while the remaining 14 genes were present in AF141925.1. When all 17 genes were aligned on the consensus sequence, it was interesting that the entire lovastatin gene cluster was detected in a single scaffold (1.16). This confirms the presence of the complete lovastatin gene cluster in *A. terreus* (KM017963).

### Nucleotide sequence accession number.

This genome sequence has been deposited at DDBJ/GenBank/EMBL under accession number LWBM00000000.
